# Association of total cerebral small vessel disease burden with left ventricular hypertrophy and geometry in patients with ischemic stroke

**DOI:** 10.3389/fnins.2026.1889756

**Published:** 2026-08-31

**Authors:** Xuanting Li, Wei Qin, Lei Yang, Shuna Yang, Yue Li, Yutong Hou, Wenli Hu

**Affiliations:** Department of Neurology, Beijing Chaoyang Hospital, Capital Medical University, Beijing, China

**Keywords:** cerebral microbleed, cerebral small vessel disease, ischemic stroke, left ventricular geometric patterns, left ventricular hypertrophy, white matter hyperintensity

## Abstract

**Purpose:**

Cerebral small vessel disease (CSVD) is a key driver of motor dysfunction, cognitive impairment, and physical frailty. Cardiac structural alterations, particularly left ventricular hypertrophy (LVH), are important prognostic markers for adverse cardiovascular outcomes. Both conditions share common risk factors, yet their clinical and pathological association remains incompletely understood.

**Methods:**

This cross-sectional study enrolled a total of 491 ischemic stroke patients. Data on the four main magnetic resonance imaging (MRI) markers of CSVD [lacune, cerebral microbleed (CMB), white matter hyperintensity (WMH), and basal ganglia perivascular space (PVS)] were collected. Based on the total CSVD MRI burden score, subjects were divided into the CSVD score 0 group (*n* = 99) and the CSVD score ≥ 1 group (*n* = 392), among whom 149 had LVH. Subjects were categorized into four left ventricular (LV) geometric patterns according to echocardiography: concentric hypertrophy, eccentric hypertrophy, concentric remodeling, and normal geometry. Binary and ordinal logistic regression analyses were performed to determine the impact of cardiac structural parameters on CSVD. Differences in CSVD markers were compared between the LVH and non-LVH groups, as well as across the four LV geometric patterns.

**Results:**

Logistic regression analysis revealed that LVH was associated with a total CSVD score of 1 or higher, and left ventricular mass index (LVMI) was positively correlated with the overall severity of CSVD. Compared with the non-LVH group, the LVH group had more CMBs, a higher total WMH score, and a higher basal ganglia PVS score, whereas no significant difference was observed in the lacune counts. Significant differences were found in the total CSVD score, total WMH score, and CMB counts across the four LV geometric patterns. The total CSVD score in the concentric hypertrophy group was higher than that in the normal geometry and concentric remodeling groups.

**Conclusion:**

LVH and elevated LVMI are associated with increased severity of CSVD in ischemic stroke patients. WMH and CMB may be influenced by LV structural alterations, and concentric hypertrophy may be closely related to CSVD. Further large-scale prospective studies are warranted to clarify the causal relationships and pathological mechanisms between CSVD and cardiac structural alterations.

## 1 Introduction

Cerebral small vessel disease (CSVD) arises from structural and functional impairments of the small blood vessels in the brain, resulting in brain parenchymal injury. The primary magnetic resonance imaging (MRI) manifestations of CSVD include recent small subcortical infarcts, white matter hyperintensity (WMH), cerebral microbleed (CMB), perivascular space (PVS), and lacune (Duering et al., 2023). The total CSVD MRI burden score offers superior utility compared with individual MRI markers for capturing the comprehensive pathological effects of CSVD (Staals et al., 2014). CSVD is highly prevalent in the aging population, accounting for approximately 25% of ischemic strokes, 85% of intracerebral hemorrhages, and the majority of vascular dementia cases (Duering et al., 2023; Wardlaw et al., 2013). It frequently coexists with neurodegenerative diseases and is closely linked to cognitive impairment, motor dysfunction, gait disturbance, and mood disorders. Despite substantial advancements in the prevention and management of atherosclerotic and cardioembolic stroke, therapeutic progress targeting CSVD remains limited.

Cardiac structural remodeling is a consequence of multiple intrinsic and extrinsic factors, encompassing physiological adaptations and pathological damage. Its major causes include hypertension, cardiac diseases, endocrine disorders, genetic and metabolic abnormalities, as well as inflammatory responses. Left ventricular hypertrophy (LVH) results from the long-term adaptation of the heart to pressure or volume overload (e.g., hypertension, obesity, valvular heart disease), characterized by cardiomyocyte hypertrophy and myocardial remodeling. It serves not only as an early marker of cardiac injury in conditions such as hypertension, obesity, and chronic kidney disease but also as an independent risk factor for cardiovascular and cerebrovascular events and all-cause mortality (Dini et al., 2019; Han et al., 2024). The prevalence of LVH ranges from 36% to 77% among patients with hypertension, diabetes mellitus, or cardiovascular and cerebrovascular diseases (Cuspidi et al., 2012; Mancusi et al., 2019).

Evidence regarding the correlation between cardiac structural alterations and CSVD remains controversial. Previous studies have shown that LVH or elevated left ventricular mass index (LVMI) may be associated with CSVD, particularly WMHs, CMBs, and lacunes (Henskens et al., 2009; Moore et al., 2018; Nakanishi et al., 2017; Papadopoulos et al., 2020; Pirinen et al., 2017). Conversely, other investigations have failed to demonstrate such an association (van der Veen et al., 2015; Cermakova et al., 2017; Görner et al., 2007). In addition, several studies have observed that left atrial volume, left ventricular (LV) wall thickness, and ejection fraction are correlated with white matter ischemia and lacunar infarction (Cermakova et al., 2017; Johansen et al., 2018).

An increasing number of studies have focused on the epidemiological associations, shared risk factors, and common pathological pathways between cardiac structural alterations and CSVD. Accordingly, the present study aimed to investigate the relationship between cardiac geometry and total CSVD burden, as well as its individual MRI markers. We hypothesized that LVH is associated with CSVD, and that this association differs across distinct LV geometric patterns. Exploring this relationship is crucial for identifying high-risk populations, elucidating multi-organ interaction pathways, and providing a theoretical basis for the prevention, treatment, and future research of cardio-cerebral axis disorders.

## 2 Materials and methods

### 2.1 Study design and participants

This cross-sectional study consecutively enrolled subjects with newly-diagnosed acute ischemic stroke who were hospitalized in the Department of Neurology, Beijing Chaoyang Hospital, Capital Medical University, between January 2022 and February 2025.

Inclusion criteria were as follows: (1) age > 18 years; (2) acute ischemic stroke with symptom onset within 1 week; (3) no contraindications to brain MRI or echocardiography; (4) ability to complete clinical data collection, brain MRI, and echocardiography; (5) provision of written informed consent by patients or their legal representative.

Exclusion criteria were: (1) evidence of intracranial hemorrhage or space-occupying lesions on brain computed tomography (CT) or MRI; (2) severe ischemic stroke or large-vessel infarction confirmed on brain CT or MRI (Hua et al., 2023); (3) white matter lesions caused by alternative etiologies, including inflammatory demyelinating diseases, central nervous system infections, traumatic brain injury, toxic-, radiation-, or metabolic-related encephalopathy; (4) patients with severe comorbidities such as cardiac, pulmonary, hepatic, or renal dysfunction; (5) suitability only for bedside echocardiography; (6) incomplete clinical data or poor-quality MRI images precluding subsequent analysis.

The study protocol was approved by the Ethics Committee of Beijing Chaoyang Hospital, Capital Medical University (Approval No. 2021-Sci-56), and was conducted in accordance with the principles of the Declaration of Helsinki.

### 2.2 Demographic and vascular risk factors

Baseline clinical data were collected within 24 h of admission, including sex, age, height, weight, blood pressure, heart rate, smoking and alcohol consumption history, past medical history [hypertension, diabetes mellitus, coronary atherosclerotic heart disease, hyperlipidemia, and stroke/transient ischemic attack (TIA)], medication history (antihypertensive agents, hypoglycemic agents, lipid-lowering agents, and antiplatelet/anticoagulant agents), and serological parameters [total cholesterol, high-density lipoprotein cholesterol (HDL-C), low-density lipoprotein cholesterol (LDL-C), triglycerides, glycated hemoglobin, and homocysteine].

#### 2.3 CSVD neuroimaging examinations

All patients underwent cranial MRI within 3 days of admission using a Prisma 3.0-T MRI system (Siemens AG, Germany). The major scanning sequences included T1-weighted imaging, T2-weighted imaging, diffusion-weighted imaging, T2-weighted fluid-attenuated inversion recovery, and susceptibility-weighted imaging.

Two neurologists, blinded to the subjects’ echocardiographic findings, independently assessed the CSVD MRI markers according to the STandards for ReportIng Vascular changes on nEuroimaging (Duering et al., 2023; Wardlaw et al., 2013). Discrepancies were resolved by consensus discussion. CMBs and lacunes of presumed vascular origin were counted separately. Basal-ganglia PVS burden was scored on a scale from 0 to 4. Deep and periventricular WMHs were separately evaluated using the Fazekas scale (0–3), and their sum yielded the total Fazekas score for WMHs (range: 0–6). Global CSVD severity was assessed using the ordinal scale of total CSVD MRI burden (0–4). One point was awarded for each of the following present markers: (1) ≥1 lacune lesion; (2) deep WMH Fazekas score ≥ 2 and/or periventricular WMH Fazekas score = 3; (3) ≥1 CMB lesion; (4) moderate-to-severe basal ganglia PVSs (scores of 2–4). Based on the score of total CSVD MRI burden, subjects were divided into the CSVD score 0 group and the CSVD score ≥ 1 group. The CSVD score ≥ 1 group was further stratified into mild (1 point), moderate (2 points), and severe (3 and 4 points) subgroups.

### 2.4 Echocardiography

All patients underwent transthoracic echocardiography within 3 days of admission using a Philips EPIQ 7C ultrasound system equipped with a 1–5 MHz S5-1 phased array probe. Two professional echocardiologists, blinded to MRI findings, performed standard plane scanning and parameter measurements in accordance with standardized technical operating procedures. Analyzed parameters included left atrial antero-posterior diameter, left atrial medio-lateral diameter, left atrial supero-inferior diameter, LV end-diastolic diameter (LVEDD), LV end-systolic diameter (LVESD), LV posterior wall thickness (LVPWT), interventricular septal thickness (IVST), right atrial medio-lateral diameter, right atrial supero-inferior diameter, and right ventricular basal diameter. All linear parameters were measured in millimeters (mm). LVMI and relative wall thickness (RWT) were calculated using the following formulas (Lang et al., 2015):


$$\text{LVMI}=\frac{\mathrm{LV}\,\mathrm{mass}}{\mathrm{body}\,\mathrm{surface}\,\mathrm{area}}$$

LVmass=0.8×1.04×[(LVEDD+IVST+

LVPWT)-3LVEDD]3+ 0.6

$$\text{RWT}=\frac{2\times \text{LVPWT}}{\text{LVEDD}}$$

All measurements were performed at end-diastole. LV mass was expressed in grams (g), and LVMI in g/m^2^.


$$\mathrm{Body}\,\mathrm{surface}\,\mathrm{area}\,\left({\text{m}}^{2}\right)=0.0061\times \mathrm{Height}\,\left(\text{cm}\right)+$$

 0.0128×Weight⁢(kg)- 0.1529

Left ventricular hypertrophy was defined as an LVMI > 115 g/m^2^ in males and >95 g/m^2^ in females. Elevated RWT was defined as RWT > 0.42 irrespective of sex. Participants were classified into four LV geometric patterns (Lang et al., 2015): normal geometry (normal LVMI and normal RWT), concentric remodeling (normal LVMI and increased RWT), concentric hypertrophy (LVH and increased RWT), and eccentric hypertrophy (LVH and normal RWT).

### 2.5 Statistical analysis

Normally distributed continuous data were presented as mean ± standard deviation, while categorical variables were presented as counts (percentages). Between-group comparisons were performed using independent-samples *t*-tests, one-way analysis of variance, and chi-square tests, as appropriate. Continuous variables with non-normal distribution and ordinal categorical data were reported as median (interquartile range). Intergroup comparisons were performed using the Mann-Whitney U test or Kruskal-Wallis H test, as appropriate. For multiple-group analyses with a significant overall difference (*P* < 0.05), Bonferroni correction was applied for *post hoc* pairwise comparisons. Variables with *P* < 0.05 in univariate analysis were enrolled in the multivariate binary logistic regression model. Binary logistic regression with the forward likelihood ratio method was performed to screen factors associated with the presence of CSVD. Furthermore, ordinal logistic regression analysis was conducted to adjust for confounding variables identified in the binary logistic regression model, to explore the independent correlation between LVMI and CSVD severity. All statistical tests were two-tailed, and a *P*-value < 0.05 was considered statistically significant. All statistical analyses were performed using IBM SPSS Statistics 26.0.

## 3 Results

**FIGURE 1:**
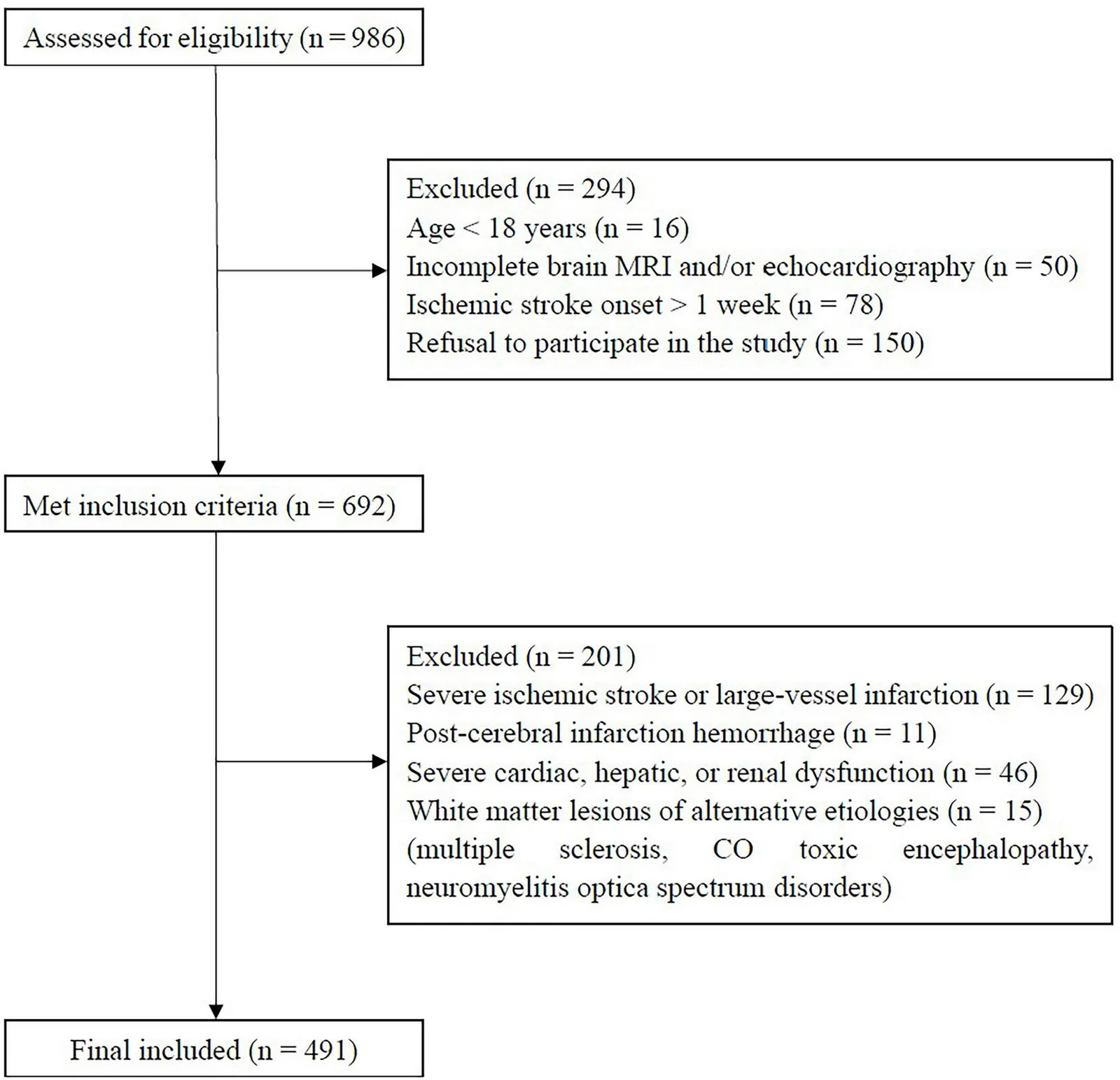
Participant screening flowchart. MRI, magnetic resonance imaging; CO, carbon monoxide.

A total of 986 patients with acute ischemic stroke were initially screened in this study. Among them, 294 patients were excluded due to ineligible age, incomplete clinical data, symptom onset exceeding 1 week, and refusal to participate. Of the remaining 692 patients who fulfilled the inclusion criteria, 201 were further excluded according to exclusion criteria: 129 with severe ischemic stroke or large-vessel infarction, 11 with hemorrhage after cerebral infarction, 46 with severe cardiac, hepatic, or renal dysfunction, and 15 with multiple sclerosis, carbon-monoxide-toxic encephalopathy, or neuromyelitis optica spectrum disorders. Ultimately, 491 participants were enrolled in the present study (344 males and 147 females; Figure 1). Among these participants, 392 were assigned to the CSVD score ≥ 1 group and 99 to the CSVD score 0 group.

The median age of the overall cohort was 68 (60, 77) years. Compared with participants in the CSVD score 0 group, those with a CSVD score ≥ 1 were significantly older (*P* < 0.001), with a higher proportion of males (*P* = 0.040), higher rates of alcohol consumption (*P* = 0.046), prior stroke/TIA (*P* < 0.001), and antiplatelet/anticoagulant agent use (*P* = 0.024), as well as a lower prevalence of diabetes mellitus (*P* = 0.047). For biochemical indicators, the CSVD score ≥ 1 group showed significantly lower levels of total cholesterol (*P* = 0.004), HDL-C (*P* = 0.016), LDL-C (*P* = 0.039), and glycated hemoglobin (*P* = 0.008), while homocysteine concentrations were significantly higher in this group (*P* = 0.001). These findings are presented in Table 1.

**TABLE 1:** Clinical characteristics of the participants.

Characteristics	Total participants (*n* = 491)	CSVD score 0 group (*n* = 99)	CSVD score ≥ 1 group (*n* = 392)	*P*
Age, years^a^	68.0 (60.0, 77.0)	64.0 (57.0, 71.0)	69.0 (61.0, 78.0)	<0.001
Male, *n* (%)	344 (70.06)	61 (61.62)	283 (72.19)	0.040
Height, cm^a^	168.00 (160.00, 172.00)	169.00 (160.00, 172.00)	168.00 (160.00, 172.00)	0.805
Weight, kg^a^	71.00 (65.00, 77.00)	71.00 (65.00, 77.00)	70.50 (65.00, 76.75)	0.329
Smoking, *n* (%)	273 (55.60)	47 (47.47)	226 (57.65)	0.069
Alcohol consumption, *n* (%)	155 (31.57)	23 (23.23)	132 (33.67)	0.046
Hypertension, *n* (%)	363 (73.93)	67 (67.68)	296 (75.51)	0.113
Diabetes mellitus, *n* (%)	234 (47.66)	56 (56.57)	178 (45.41)	0.047
Coronary heart disease, *n* (%)	127 (25.87)	23 (23.23)	104 (26.53)	0.503
Stroke/TIA, *n* (%)	219 (44.60)	19 (19.19)	200 (51.02)	<0.001
Hyperlipidemia, *n* (%)	123 (25.05)	23 (23.23)	100 (25.51)	0.640
Antihypertensive drugs, *n* (%)	311 (63.34)	57 (57.58)	254 (64.80)	0.183
Antiplatelet/anticoagulant drugs, *n* (%)	150 (30.55)	21 (21.21)	129 (32.91)	0.024
Hypoglycemic drugs, *n* (%)	210 (42.77)	49 (49.49)	161 (41.07)	0.130
Lipid-lowering drugs, *n* (%)	98 (19.96)	16 (16.16)	82 (20.92)	0.290
Total cholesterol, mmol/L^b^	4.54 ± 1.08	4.82 ± 1.11	4.46 ± 1.06	0.004
HDL-C, mmol/L^a^	1.01 (0.90, 1.20)	1.10 (0.91, 1.30)	1.00 (0.86, 1.20)	0.016
LDL-C, mmol/L^a^	2.70 (2.05, 3.33)	3.00 (2.28, 3.50)	2.60 (2.00, 3.30)	0.039
Glycated hemoglobin, %	6.50 (5.80, 8.20)	7.30 (6.00, 9.50)	6.50 (5.73, 8.00)	0.008
Homocysteine, μmol/L^a^	17.00 (13.00, 21.00)	15.00 (11.00, 20.00)	17.00 (13.00, 21.00)	0.001
Systolic blood pressure, mmHg^a^	150.00 (137.00,165.00)	150.00 (137.00, 162.00)	150.00 (137.00, 165.00)	0.751
Diastolic blood pressure, mmHg^a^	81.00 (73.00, 90.00)	80.00 (73.00, 90.00)	81.50 (73.00, 90.00)	0.719
Heart rate, beats/min^a^	76.00 (70.00, 82.00)	76.00 (70.00, 84.00)	76.00 (70.00, 82.00)	0.862
LVEF, %	67.00 (63.00, 71.00)	67.00 (64.00, 73.00)	67.00 (63.00, 70.75)	0.418

CSVD, cerebral small vessel disease; TIA, transient ischemic attack; HDL-C, high-density lipoprotein cholesterol; LDL-C, low-density lipoprotein cholesterol; LVEF, left ventricular ejection fraction.

^a^Median (interquartile range).

^b^Mean (standard deviation).

For cardiac structural parameters, LVPWT (*P* < 0.001), IVST (*P* = 0.005), RWT (*P* = 0.019), and LVMI (*P* < 0.001) were all significantly higher in the CSVD score ≥ 1 group than in the CSVD score 0 group (Table 2). No significant intergroup differences were observed for LVEDD, LVESD, left atrial dimensions, right ventricular basal diameter, or right atrial dimensions (all *P* > 0.05).

**TABLE 2:** Comparison of cardiac structural parameters between the CSVD score 0 group and the CSVD score ≥ 1 group.

Parameters^a^	Total participants (*n* = 491)	CSVD score 0 group (*n* = 99)	CSVD score ≥ 1 group (*n* = 392)	*P*
LVEDD, mm	46.00 (44.00, 49.00)	46.00 (43.00, 49.00)	47.00 (44.00, 49.00)	0.114
LVESD, mm	28.00 (25.00, 31.00)	28.00 (25.00, 31.00)	28.00 (26.00, 31.00)	0.608
LVPWT, mm	10.00 (9.00, 11.00)	10.00 (9.00, 10.00)	10.00 (9.00, 11.00)	<0.001
LAAPD, mm	37.00 (34.00, 40.00)	37.00 (35.00, 40.00)	37.00 (34.00, 40.75)	0.646
LAMLD, mm	38.00 (35.00, 42.00)	38.00 (34.00, 42.00)	38.00 (36.00, 42.00)	0.307
LASID, mm	50.00 (47.00, 55.00)	50.00 (46.00, 54.00)	51.00 (47.00, 55.00)	0.199
IVST, mm	10.50 (9.80, 12.00)	10.00 (9.00, 11.00)	11.00 (9.93, 12.00)	0.005
RVBD, mm	32.00 (30.00, 35.00)	33.00 (29.50, 34.00)	32.00 (30.00, 35.00)	0.885
RAMLD, mm	33.00 (29.50, 37.00)	33.00 (33.00, 36.00)	33.00 (29.00, 37.00)	0.599
RASID, mm	45.00 (41.00, 48.50)	45.00 (42.00, 47.00)	45.00 (41.00, 49.75)	0.957
RWT	0.43 (0.39, 0.48)	0.42 (0.38, 0.46)	0.43 (0.39, 0.48)	0.019
LVMI, g/m^2^	96.89 (83.03, 111.83)	90.72 (76.79, 102.59)	98.42 (84.96, 113.94)	<0.001

CSVD, cerebral small vessel disease; LVEDD, left ventricular end-diastolic diameter; LVESD, left ventricular end-systolic diameter; LVPWT, left ventricular posterior wall thickness; LAAPD, left atrial antero-posterior diameter; LAMLD, left atrial medio-lateral diameter; LASID, left atrial supero-inferior diameter; IVST, interventricular septal thickness; RVBD, right ventricular basal diameter; RAMLD, right atrial medio-lateral diameter; RASID, right atrial supero-inferior diameter; RWT, relative wall thickness; LVMI, left ventricular mass index.

^a^Median (interquartile range).

A binary logistic regression model was constructed incorporating age, sex, alcohol consumption, diabetes mellitus, prior stroke/TIA, antiplatelet/anticoagulant agent use, glycated hemoglobin, total cholesterol, HDL-C, LDL-C, homocysteine, IVST, LVPWT, LVMI, and RWT. The results demonstrated that age (odds ratio [OR] = 1.05, 95% confidence interval [CI]: 1.02–1.07, *P* < 0.001), prior stroke/TIA (OR = 4.55, 95% CI: 2.57–8.03, *P* < 0.001), HDL-C (OR = 0.37, 95% CI: 0.16–0.85, P = 0.019), glycated hemoglobin (OR = 0.87, 95% CI: 0.78–0.97, *P* = 0.015), and LVMI (OR = 1.02, 95% CI: 1.01–1.03, *P* = 0.003) were independently associated with a total CSVD score of 1 or higher. Detailed results are summarized in Table 3.

**TABLE 3:** Binary logistic regression analysis of independent risk factors of CSVD.

Variables	OR	95% CI	*P*
Age	1.05	1.02, 1.07	<0.001
Stroke/TIA	4.55	2.57, 8.03	<0.001
Glycated hemoglobin	0.87	0.78, 0.97	0.015
HDL-C	0.37	0.16, 0.85	0.019
LVMI	1.02	1.01, 1.03	0.003

CSVD, cerebral small vessel disease; TIA, transient ischemic attack; HDL-C, high-density lipoprotein cholesterol; LVMI, left ventricular mass index; OR, odds ratio; 95% CI, 95% confidence interval.

A total of 149 participants had LVH, of whom 80 were male, and 134 had total CSVD scores of 1 or higher. After adjustment for age, sex, alcohol consumption, diabetes mellitus, prior stroke/TIA, antiplatelet/anticoagulant agent use, glycated hemoglobin, total cholesterol, HDL-C, LDL-C, homocysteine, IVST, LVPWT, and RWT, binary logistic regression showed that LVH was positively associated with a total CSVD score ≥ 1 (OR = 2.77, 95% CI: 1.45–5.30, *P* = 0.002).

Among the 392 participants with a total CSVD score ≥ 1, 116 were classified into the mild subgroup, 108 into the moderate subgroup, and 168 into the severe subgroup. Age, prior stroke/TIA, glycated hemoglobin, HDL-C, and LVMI were entered into the ordinal logistic regression model, which satisfied the proportional-odds assumption (*P* = 0.091). The results demonstrated that the global severity of CSVD increased with elevated LVMI (OR = 1.01, 95% CI: 1.00–1.02, *P* = 0.004). LV structural parameters stratified by CSVD severity are presented in Table 4.

**TABLE 4:** Comparison of left ventricular parameters across different CSVD severity subgroups.

Parameters^a^	CSVD score 0 group (*n* = 99)	Mild CSVD subgroup (*n* = 116)	Moderate CSVD subgroup (*n* = 108)	Severe CSVD subgroup (*n* = 168)	H	*P*
LVEDD, mm	46.00 (43.00, 49.00)	47.00 (44.00, 49.00)	46.00 (44.00, 50.00)	47.00 (44.00, 49.00)	2.72	0.437
LVESD, mm	28.00 (25.00, 31.00)	28.00 (26.00, 31.00)	28.00 (26.00, 31.00)	28.00 (25.00, 31.00)	3.70	0.295
LVPWT, mm	10.00 (9.00, 10.00)	10.00 (9.00, 11.00)	10.00 (9.00, 11.00)	10.00 (9.00, 11.00)	14.55	0.002
IVST, mm	10.00 (9.00, 11.00)	10.60 (9.43, 12.00)	10.70 (9.45, 12.00)	11.00 (10.00, 12.00)	13.23	0.004
RWT	0.42 (0.38, 0.46)	0.43 (0.39, 0.48)	0.44 (0.39, 0.48)	0.43 (0.40, 0.48)	6.50	0.090
LVMI, g/m^2^	90.72 (76.79, 102.59)	98.02 (86.22, 114.92)	96.83 (83.35, 112.04)	99.93 (86.18, 117.17)	14.45	0.002

CSVD, cerebral small vessel disease; LVEDD, left ventricular end-diastolic diameter; LVESD, left ventricular end-systolic diameter; LVPWT, left ventricular posterior wall thickness; IVST, interventricular septal thickness; RWT, relative wall thickness; LVMI, left ventricular mass index.

^a^Median (interquartile range).

Compared with the non-LVH group, the LVH group had a significantly higher total CSVD score (*Z* = −2.94, *P* = 0.003), more CMB lesions (*Z* = −2.16, *P* = 0.031), a higher total WMH score (*Z* = −3.06, *P* = 0.002), and a higher basal-ganglia PVS score (*Z* = −2.36, *P* = 0.018). No statistically significant difference was observed in the number of lacunes between the two groups (*Z* = −1.17, *P* = 0.244) (Table 5).

**TABLE 5:** Comparison of CSVD MRI markers among different cardiac structure groups.

Groups	CSVD score ≥ 1, n (%)	Total CSVD score^a^	Lacunes^a^	CMBs^a^	Total WMH score^a^	Basal-ganglia PVS score^a^
Non-LVH group (*n* = 342)	258 (75.44)	2 (1, 3)	1 (0, 2)	0 (0, 1)	3 (2, 4)	1 (1, 2)
LVH group (*n* = 149)	134 (89.93)	2 (1, 3)	1 (0, 2)	0 (0, 2.5)	4 (2, 4)	1 (1, 2)
*P*	<0.001	0.003	0.244	0.031	0.002	0.018
Normal geometry (*n* = 171)	126 (73.68)	2 (0, 3)	1 (0, 2)	0 (0, 1)	2 (2, 4)	1 (1, 2)
Concentric remodeling (*n* = 171)	132 (77.19)	2 (1, 3)	1 (0, 2)	0 (0, 1)	3 (2, 4)	1 (1, 2)
Concentric hypertrophy (*n* = 105)	97 (92.38)	2 (1, 3)	1 (0, 2)	0 (0, 3)	4 (2, 4)	1 (1, 2)
Eccentric hypertrophy (*n* = 44)	37 (84.09)	2 (1, 3)	0.5 (0, 2)	0 (0, 1)	4 (2, 5)	1.5 (1, 2.75)
*P*	0.001	0.019	0.268	0.045	0.015	0.070

CSVD, cerebral small vessel disease; MRI, magnetic resonance imaging; CMBs, cerebral microbleeds; WMH, white matter hyperintensity; PVS, perivascular space; LVH, left ventricular hypertrophy.

^a^Median (interquartile range).

When participants were stratified according to LV geometric patterns, the prevalence of CSVD differed significantly among the four groups (χ^2^ = 15.52, *P* = 0.001). The concentric hypertrophy group exhibited the highest proportion of participants with a total CSVD score of 1 or higher (92.38%), whereas the normal geometry group had the highest proportion of participants with a total CSVD score of 0 (26.32%). Significant differences were showed in the total CSVD score (H = 9.94, *P* = 0.019), total WMH score (H = 10.52, *P* = 0.015), and CMB counts (H = 8.06, *P* = 0.045) across the four groups. In contrast, no significant differences were found in lacune counts (H = 3.94, *P* = 0.268) or basal-ganglia PVS scores (H = 7.07, *P* = 0.070) (Table 5). *Post hoc* pairwise comparisons demonstrated that the total CSVD score was significantly higher in the concentric hypertrophy group than in both the normal geometry group (adjusted *P* = 0.027) and the concentric remodeling group (adjusted *P* = 0.032). Furthermore, the total WMH score in the concentric hypertrophy group was significantly higher than that in the normal geometry group (adjusted *P* = 0.013), and the number of CMBs in the concentric hypertrophy group was higher than that in the concentric remodeling group (adjusted *P* = 0.028). No significant differences in CSVD MRI markers were identified in any other pairwise comparisons.

## 4 Discussion

In the present study, LVH was significantly associated with a total CSVD score of 1 or higher, and global CSVD severity increased with elevated LVMI. Compared with the non-LVH group, the LVH group had a higher total CSVD score, a greater number of CMBs, a higher total WMH score, and a higher basal-ganglia PVS score, whereas no significant difference was observed in the number of lacunes between the two groups. CSVD prevalence differed across the four LV geometric pattern subgroups: the concentric hypertrophy group had the highest proportion of patients with a CSVD score of 1 or higher, while the normal geometry group had the highest proportion of individuals with a CSVD score of 0. Significant differences were found in the total CSVD score, total WMH score, and CMB counts across the four subgroups. *Post hoc* analyses further revealed that the concentric hypertrophy group had higher total CSVD scores relative to both the normal geometry group and the concentric remodeling group; higher total WMH scores compared with the normal geometry group; and greater CMB counts versus the concentric remodeling group.

Previous studies have proposed a potential close relationship between LVH or elevated LVMI and CSVD, which is consistent with our present findings. LVMI has been reported to correlate with asymptomatic CSVD, including WMHs, CMBs, lacunes, or any combination of these lesions (Henskens et al., 2009). One recent study demonstrated that patients with concentric hypertrophy exhibited a higher prevalence of substantial CSVD burden and dementia relative to individuals with the other three LV geometric patterns (Huang et al., 2025). Fabry disease, a hereditary CSVD subtype, frequently manifests with LVH as its predominant cardiac feature. Electrocardiographic and cardiac-MRI abnormalities may emerge at an early stage, reflecting a prolonged “pre-hypertrophic” cardiac phase (Geenty and Thomas, 2024). However, conflicting evidence has shown no significant differences in the prevalence of left atrial enlargement and LVH between patients with a total CSVD score of 0–2 and those with a score of 3–4, suggesting that left cardiac structural changes may not be associated with the total CSVD burden (Gómez-Choco et al., 2023).

Conclusions concerning the association between LVH and individual CSVD MRI markers remain inconsistent. Accumulating evidence has shown that LVH or elevated LVMI is significantly associated with higher WMH scores or larger lesion volumes (Johansen et al., 2018; Moore et al., 2018, 2022; Nakanishi et al., 2017; Salem et al., 2024; Vedala et al., 2019), and elevated LVMI correlates with impaired white matter microstructure in the elderly (Moore et al., 2018). Other studies have suggested that LVH is associated only with deep WMHs, but not periventricular WMHs (Nagaraja et al., 2022). Additionally, several studies have found that higher LVMI is linked to CMBs in the whole brain, brainstem and subcortical white matter, supporting a close relationship between CMBs and LVH (Henskens et al., 2009; Lee et al., 2004). Likewise, our study found that the LVH group had a greater number of CMBs and a higher total WMH score compared with the non-LVH group. Previous research has indicated that LVH may be associated with lacunes (Nakanishi et al., 2017; Pirinen et al., 2017), while no significant difference in lacune counts was observed between the LVH and non-LVH groups in our study. In contrast, several other studies have reported that LVH or elevated LVMI is not significantly correlated with CMBs, WMHs, or lacunar infarcts (Cermakova et al., 2017; Görner et al., 2007; van der Veen et al., 2015). A meta-analysis of 31 studies has found that LVH was associated with lacunar infarction, extensive WMHs, and CMBs; after adjusting for age, sex, hypertension, and other vascular risk factors, the association between LVH and lacunes/WMHs remained stable (Papadopoulos et al., 2020). However, this meta-analysis did not assess total CSVD MRI burden or include eligible studies assessing PVS, which limits its capacity to provide a comprehensive understanding of the relationship between LVH and overall CSVD severity. The present study found that the LVH group had higher basal-ganglia PVS scores than the non-LVH group, but no significant difference in PVS scores was observed among different LV geometric patterns. This finding highlights the need for further investigations to validate this association.

In addition, one study has suggested that larger left atrial volume is associated with lower fractional anisotropy of cerebral white matter, and this correlation is strongest in Black and male participants (Cermakova et al., 2017). The Atherosclerosis Risk in Communities Study has shown that LV wall thickness is positively correlated with WMH severity and also increases the risk of subclinical lacunar infarction. Furthermore, a higher ejection fraction was significantly associated with decreased odds of overall infarcts (Johansen et al., 2018). Another study on the mechanism of non-traumatic intracerebral hemorrhage found that the absence of LVH is a protective factor for mixed intracerebral hemorrhage (with CMBs in different locations and/or intracerebral hemorrhage) complicated by cortical superficial siderosis (Das et al., 2023). However, the results of the present study showed no significant differences in left atrial size, right atrial size, or ejection fraction between the CSVD score 0 group and the CSVD score ≥ 1 group. Although LVPWT and RWT were higher in the CSVD score ≥ 1 group than in the CSVD score 0 group, no significant correlation was observed after adjusting for confounding factors.

Different LV geometric patterns may have distinct associations with CSVD. One study found that concentric hypertrophy and eccentric hypertrophy were associated with asymptomatic lacunes and WMHs, whereas concentric remodeling showed no correlation with subclinical CSVD (Nakanishi et al., 2017). Compared with eccentric hypertrophy and normal geometry, patients with concentric hypertrophy and concentric remodeling had larger WMH volumes (Salem et al., 2024). Another study demonstrated that both concentric remodeling and concentric hypertrophy were significantly correlated with an increased number of cortical cerebral microinfarcts, while eccentric hypertrophy showed no such association. A significant association was also observed between concentric hypertrophy and both the number of cortical cerebral microinfarcts and the odds of dementia; moreover, cortical cerebral microinfarcts may partly explain the relationship between concentric hypertrophy and cognitive function (Huang et al., 2025). Concentric hypertrophy is associated with the worst prognosis in patients with hypertension and coronary artery disease (Dini et al., 2019). Therefore, it may serve as a key target for future research exploring the pathophysiological mechanisms underlying the impact of cardiac structural remodeling on CSVD. Detailed associations between individual CSVD MRI markers and LV features deserve further exploration in more studies.

The heart-brain homology theory posits that cardiovascular and cerebrovascular diseases share common risk factors, such as hypertension, diabetes mellitus, obesity, and atherosclerosis. Chronic hypertension can induce myocardial injury, cardiac remodeling, and cerebral small vessel damage (Hainsworth et al., 2024; Han et al., 2024; Markus and Joutel, 2025), while metabolic disorders may accelerate atherosclerotic progression (Virani et al., 2021; Xue et al., 2023; Zhang et al., 2022), thereby contributing to the development of cardio-cerebrovascular diseases. LVH may lead to abnormal hemodynamic changes characterized by reduced cardiac output, ultimately impairing cardiac reserve (Fang et al., 2025; Romejko et al., 2022). Different LV geometric patterns may also be associated with distinct hemodynamic characteristics: compared to subjects with normal geometry or concentric remodeling, patients with LVH exhibit higher peripheral vascular resistance and poorer systolic and diastolic function (Chahal et al., 2010). These adverse hemodynamic features may compromise cardiac functional efficiency and alter cerebral perfusion. Hemodynamic disturbances and cerebral hypoperfusion are recognized as critical factors contributing to the pathogenesis and progression of CSVD markers such as lacunar infarcts, WMH, and lacunes (Hainsworth et al., 2024; Markus and Joutel, 2025). Cardiac dysfunction secondary to LV structural alterations may trigger a cascade of pathological processes, including cardiac arrhythmias, cardiogenic embolism, and venous congestion, leading to cerebral ischemia and inflammation, which ultimately result in cortical cerebral microinfarcts and other CSVD subtypes (Huang et al., 2025). Furthermore, inflammatory responses and oxidative stress are likely to represent critical molecular mechanisms underlying the structural and functional interplay between LVH and CSVD (Bacharova et al., 2023; Fang et al., 2025; Hainsworth et al., 2024). Additionally, a separate study has demonstrated that axonal injury may partially mediate the association between increased LVMI and cerebral white matter damage (Moore et al., 2022). However, previous research has yielded inconsistent conclusions regarding the correlation between LVH/elevated LVMI and various CSVD MRI markers, suggesting that the mechanisms underlying the impact of cardiac structure and function on different CSVD subtypes may be heterogeneous.

Some studies have confirmed that apolipoprotein E (APOE) polymorphisms are closely associated with both cardiac structural remodeling and CSVD. The APOE ε4 allele is the strongest known high-risk genetic variant for cerebrovascular diseases (Tai et al., 2016), and is also related to cardiac remodeling and elevated heart failure risk (Jung et al., 2021; Liu et al., 2022; Wang et al., 2024). The APOE ε2 allele improves cardiomyocyte contractility and preserves cardiac function (Jung et al., 2021; Wang et al., 2024), yet it confers higher susceptibility to cortical superficial siderosis and lobar intracerebral hemorrhage (Bhagat et al., 2023). Given the modulatory effects of APOE variants on the cardio-cerebral axis diseases, well-designed prospective cohort studies are warranted to incorporate genetic biomarkers and further elucidate the shared genetic mechanisms underlying concurrent cardiac structural abnormalities and CSVD.

Few studies have explored the relationship between LV geometric patterns and total CSVD MRI burden. The present study not only identified an association between global CSVD severity and LVH/elevated LVMI in patients with ischemic stroke but also conducted a preliminary analysis of the relationship between individual CSVD MRI markers and LV structural abnormalities. However, several limitations of this study should be acknowledged. Firstly, as a cross-sectional study, it cannot establish a temporal or causal relationship between CSVD and cardiac structural alterations. Secondly, this study was conducted in patients with ischemic stroke, limiting the generalizability of the findings to other populations. Finally, total CSVD burden and individual MRI markers were assessed using semi-quantitative methods; future studies should utilize multimodal imaging techniques to provide a more comprehensive assessment of CSVD.

Interventions such as antihypertensive therapy, weight reduction, lifestyle modifications, and surgical approaches can decrease LVMI and attenuate LVH in some patients, suggesting the reversibility of cardiac structural alterations (Sesa-Ashton et al., 2025; Tatavarthy et al., 2024). If early intervention in cardiac remodeling could slow the progression of CSVD, it would be of great significance for reducing the risks of cerebral infarction, cognitive impairment, and mortality. However, multicenter, large-scale, prospective cohort studies and interventional trials are needed to provide robust evidence for the comprehensive prevention and treatment of cardio-cerebral axis diseases.

## 5 Conclusion

Left ventricular hypertrophy and elevated LVMI are independently related to the presence and increased severity of CSVD in patients with ischemic stroke. Among CSVD markers, WMH and CMB may be more susceptible to LV structural alterations. Concentric hypertrophy may be more closely associated with CSVD compared with the other three LV geometric patterns. More studies are needed to further clarify the causal relationship and pathological mechanisms linking CSVD to cardiac structural remodeling to facilitate the identification of high-risk patients and individualized interventions.

## Data Availability

The raw data supporting the conclusions of this article will be made available by the authors, without undue reservation.
